# Clinical applications of radiomics in non-small cell lung cancer patients with immune checkpoint inhibitor-related pneumonitis

**DOI:** 10.3389/fimmu.2023.1251645

**Published:** 2023-09-20

**Authors:** Yang Shu, Wei Xu, Rui Su, Pancen Ran, Lei Liu, Zhizhao Zhang, Jing Zhao, Zhen Chao, Guobin Fu

**Affiliations:** ^1^ Department of Oncology, Shandong Provincial Hospital Affiliated to Shandong First Medical University, Jinan, Shandong, China; ^2^ The Second Clinical Medical College, Shandong University of Traditional Chinese Medicine, Jinan, Shandong, China; ^3^ Department of Oncology, Shandong Provincial Hospital, Shandong University, Jinan, Shandong, China; ^4^ College of Artificial Intelligence and Big Data for Medical Sciences, Shandong First Medical University and Shandong Academy of Medical Sciences, Jinan, Shandong, China; ^5^ Department of Oncology, The Third Affiliated Hospital of Shandong First Medical University, Jinan, Shandong, China

**Keywords:** radiomics, non-small cell lung cancer, immune checkpoint inhibitor-related pneumonitis, deep learning, immunotherapy

## Abstract

Immune checkpoint inhibitors (ICIs) modulate the body’s immune function to treat tumors but may also induce pneumonitis. Immune checkpoint inhibitor-related pneumonitis (ICIP) is a serious immune-related adverse event (irAE). Immunotherapy is currently approved as a first-line treatment for non-small cell lung cancer (NSCLC), and the incidence of ICIP in NSCLC patients can be as high as 5%-19% in clinical practice. ICIP can be severe enough to lead to the death of NSCLC patients, but there is a lack of a gold standard for the diagnosis of ICIP. Radiomics is a method that uses computational techniques to analyze medical images (e.g., CT, MRI, PET) and extract important features from them, which can be used to solve classification and regression problems in the clinic. Radiomics has been applied to predict and identify ICIP in NSCLC patients in the hope of transforming clinical qualitative problems into quantitative ones, thus improving the diagnosis and treatment of ICIP. In this review, we summarize the pathogenesis of ICIP and the process of radiomics feature extraction, review the clinical application of radiomics in ICIP of NSCLC patients, and discuss its future application prospects.

## 1 Introduction

Currently, lung cancer remains the leading cause of cancer-related deaths, with approximately over 20% of cancer patients dying from lung cancer (1, 2). Non-small cell lung cancer (NSCLC) accounts for 85% of all lung cancer cases (3), and it primarily includes adenocarcinoma and squamous cell carcinoma (4). Approximately 70% of NSCLC patients are diagnosed at an advanced stage (5), and for advanced NSCLC, a comprehensive treatment approach is typically employed, including radiation therapy, chemotherapy, targeted therapy, and immunotherapy (6). Immunotherapy has emerged as a novel treatment method and has gained significant attention due to its ability to improve the overall survival and survival rates of patients with NSCLC (7–9). Immunotherapy has made significant progress in advanced NSCLC patients, with an unselected 5-year overall survival rate (OS) of 20%, and a 5-year overall survival rate of up to 40% in patients with high PD-L1 expression (10, 11). However, while immunotherapy enhances patient survival, it can also give rise to a series of immune-related adverse events (irAEs) that involve the skin, respiratory system, digestive system, and cardiovascular system (12–14). Immune checkpoint inhibitor-related pneumonitis (ICIP) is the most common adverse respiratory reaction caused by immunotherapy (15), and it is also the primary cause of death resulting from immune-related adverse events (16).

ICIP refers to the development of newly observed pulmonary inflammatory infiltrates on medical imaging after the initiation of immunotherapy, excluding other causes of pneumonitis during the progression of cancer. A retrospective analysis showed that the overall incidence of pneumonitis in cancer patients receiving treatment with anti-programmed cell death-1/programmed cell death ligand-1 (anti-PD-1/PD-L1) monoclonal antibodies or in combination with anti-cytotoxic T-cell lymphocyte-4 monoclonal antibody was 5% (3%-6%). The incidence of pneumonitis was higher with combination immunotherapy compared to monotherapy (17). Another systematic review and meta-analysis of studies on melanoma, NSCLC, and renal cell carcinoma (RCC) showed that ICIP is common in NSCLC,renal cell carcinoma, and during combination therapy (18). In clinical trials, the incidence of ICIP in NSCLC is generally 3% to 5% (19, 20), but in the real clinical application environment, the incidence of ICIP in NSCLC can range from 5% to 19% (15, 21–23).

Immune-related adverse events (irAEs), including ICIP, can have a certain impact on the treatment plan, prognosis, and treatment cost of patients with NSCLC. During the progression of NSCLC, in addition to pneumonitis caused by immune therapy, infections (24), radiotherapy (25), chemotherapy (26), and targeted therapy (27) can also induce pneumonitis. Currently, there is no established gold standard for diagnosing ICIP, and clinicians usually make subjective inferences based on patient symptoms and imaging findings (28). Diagnostic methods such as imaging characteristics, clinical risk factors, bronchoalveolar lavage, and cell culture lack specificity for diagnosing ICIP. The concept of radiomics was first proposed by Professor Philippe Lambin in 2012 (29), was later elaborated upon by Kumar et al. (30). It refers to the high-throughput extraction and analysis of advanced imaging features from medical images such as CT, PET, or MRI. Radiomics utilizes high-throughput data extraction techniques to identify features in non-invasive imaging data and establish predictive models, solving classification and regression challenges in clinical practice (31). By using radiomics models, relevant features can be screened from medical imaging data, transforming qualitative clinical problems into quantitative ones, thus providing a more objective method for solving clinical problems (32). In recent years, radiomics has been used for the diagnosis, pathological classification, clinical staging, efficacy assessment, and prognosis evaluation of NSCLC (33–36). With the development of precision medicine, radiomics can further be used for predicting, diagnosing, differentiating and prognostic analysis ICIP in NSCLC patients. This can enable close monitoring and guide the diagnosis and treatment of ICIP in NSCLC patient. This paper reviews the pathogenesis of ICIP and the process of radiomics feature extraction. It aims to conduct a comprehensive analysis of the clinical studies applying radiomics to NSCLC patients with ICIP. In addition, this review discusses the limitations of these studies and proposes potential approaches to address them.

## 2 Immunotherapy and ICIP in patients with NSCLC

Currently, immune checkpoint inhibitors (ICIs) used for NSCLC mainly include Nivolumab, Pembrolizumab, Cemiplimab, Atezolizumab, Avelumab, Durvalumab, and Ipilimumab (37, 38). In addition to monotherapy, ICIs can also be used in combination with chemotherapy drugs, targeted drugs, and anti-angiogenic drugs (39). The overall response rate of immune therapy in NSCLC is approximately 20-30% (40). A phase 1b multicenter trial showed that the combination of Durvalumab and Osimertinib in the treatment of EGFR mutation-positive NSCLC patients can increase the incidence of interstitial lung disease (ILD)-related adverse events (AE) (35%) (41). ICIs have been a hot topic in cancer treatment research in recent years, primarily targeting programmed cell death-1 (PD-1) or programmed cell death ligand-1 (PD-L1) and cytotoxic T-lymphocyte-associated antigen-4 (CTLA-4) in the body’s immune process (42). PD1/PD-L1 primarily suppresses immune responses by inhibiting intracellular signal transduction in effector T cells and regulatory T cells, mainly by reducing the activity of phosphoinositide 3-kinase (PI3K) (43, 44). Cytotoxic T-lymphocyte-associated antigen-4 (CTLA-4) and CD28 have opposing immune regulatory functions, with CTLA4 binding more easily to B7 than CD28, thus preventing CD28 from binding to B7 and inhibiting CD28-mediated T cell proliferation process (45, 46). The mechanism of ICIP is still not fully understood,but ICIs can induce immune dysregulation while targeting tumor cells. Immune dysregulation mainly involves T-cell imbalance, increased production of autoantibodies, and dysregulation of inflammatory cytokines (47). Following anti-PDL1/PD1 immune therapy, infiltration of Th1 cells [most of which express PD-1 (48)] can be observed in corresponding tissues (49, 50). Immune therapy limits the conversion of Th1 cells into Treg cells (51), and a decrease in Treg cells may lead to immune dysfunction. Anti-PD-1 therapy can enhance the anti-tumor function of Th17 cells (52, 53). ICIP may also be associated with autoantibodies, as studies have shown an increase in pre-existing and newly developed autoantibodies following immune checkpoint inhibitor treatment (54, 55). Inflammatory markers such as C-reactive protein (CRP) and IL-6 can induce excessive activation of the immune system (56). The serum levels of IL-17 are increased in patients with NSCLC who develop ICIP after immunotherapy (57). The potential risk factors for ICIP include patient baseline characteristics, disease features, and treatment management (58). These risk factors have a high correlation with ICIP and can be combined with radiomics features to build multimodal models (59, 60).

## 3 Screening for radiomic features of ICIP

ICIP essentially refers to an interstitial inflammation. The radiological manifestations of ICIP are similar to those of conventional pneumonia, including ground-glass opacities, consolidations, fibrous bands, thickening of interlobular septa, traction bronchiectasis, small nodular opacities, and reticular opacities (61–63). ICIP can be classified based on its radiological manifestations, including cryptogenic organizing pneumonia (COP), non-specific interstitial pneumonia (NSIP), acute interstitial pneumonia/acute respiratory distress syndrome (AIP/ARDS), and hypersensitivity pneumonitis (HP) (64). ICIP does not exhibit specific features in chest medical imaging pictures (61), and even radiologists with decades of experience cannot visually differentiate ICIP with the naked eye. The heterogeneity of tumor cells at the cellular level is closely related to the radiological features present in medical imaging data (65). Radiomics employs computer algorithms to extract high-throughput information from medical imaging data and transform qualitative data into quantitative data (32). Radiomics is divided into conventional radiomics and deep learning-based radiomics.

### 3.1 Conventional radiomics feature selection

Conventional radiomics requires manual extraction and selection of radiomics features. Conventional radiomics features include size, shape, and texture features, with texture features further divided into first-order, second-order, and higher-order texture features (66, 67). Extracting too many radiomics features can lead to model overfitting, hence the need to screen hundreds or thousands of features (68). Common conventional radiomics feature selection methods include the Least Absolute Shrinkage and Selection Operator (LASSO) (69), Principal Component Analysis (PCA) (70), and Minimum Redundancy Maximum Relevance (MRMR) (71). Conventional radiomics methods may overlook higher-level features when extracting radiomics features.

### 3.2 Deep learning radiomics feature selection

Research has shown that deep learning features are typically more representative and robust than manually designed traditional radiomics features (72). Deep learning radiomics, using architectures like CNNs and Transformers, employ forward propagation for computations and data transformations, yielding predictions. The network’s training hinges on backpropagation, which calculates gradients based on prediction error. These gradients iteratively refine the network’s parameters. This dynamic between forward and backpropagation enables automatic extraction of radiomics features, revealing advanced radiomics traits.

#### 3.2.1 CNNs

CNNs are the most classical deep neural network architecture. GoogLeNet/Inception, ResNet, VGGNet, and DenseNet are commonly used CNN models in lung cancer radiomics (73). VGGNet (74) are known for their simplicity, using only 3x3 convolutions to build deep networks, which improved accuracy. GoogLeNet/Inception (75) deepens network architectures and uniquely extracts and merges multi-scale image features using its Inception modules, optimizing computational efficiency without compromising performance. However, both VGGNet and GoogLeNet/Inception encounter a common challenge: the potential for vanishing or exploding gradients as the CNN architecture deepens. This can lead to suboptimal weight updates in earlier layers, consequently diminishing the model’s performance. ResNet (76) employs Batch Normalization and skip connections to address the vanishing or exploding gradient problem. This approach effectively mitigates the performance degradation associated with internal covariate shift, which arises from scale variations in input data and layer computations. DenseNet (77), building upon ResNet’s advantages, introduces dense connectivity for enhanced feature reuse and gradient flow. This achieves parameter efficiency and superior performance. It employs a ‘growth rate’ for layer size and transition layers for feature-map management. However, dense connections elevate computational demands and memory usage. Issues like feature redundancy and increased complexity can arise. Kundu et al. developed an integrated model for the early diagnosis of pneumonia using GoogLeNet, ResNet-18, and DenseNet-121 (78). The model was evaluated using chest X-ray images from the Kermany dataset and the RSNA dataset, achieving accuracies of 98.81% and 86.86%, respectively.

#### 3.2.2 Transformers

The Transformer, originally designed for Natural Language Processing (NLP) tasks, has been adapted for computer vision, capturing intricate contextual relationships within images. In some applications, especially medical image representation, Transformer-based models have demonstrated competitive, if not superior, performance compared to traditional CNNs (79–82). Swin Transformer is a novel Transformer model proposed by Ze Liu et al. in 2021. This model is a hierarchical vision transformer using shifted windows, which enables the application of Transformers from the language domain to the visual domain (83). Ma, X et al. (84) retrospectively collected CT data from 612 patients with lung adenocarcinoma, which were divided into a training set (n=189), an internal validation set (n=123), and an external test set (n=108). In this study, deep learning model based on Swin Transformer, conventional radiomics model, and clinical semantic model were employed to predict lymph node metastasis. The results showed that the deep learning model based on Swin Transformer outperformed both clinical semantic model and conventional radiomics model in all three datasets.

## 4 Radiomics application in patients with NSCLC who have ICIP

### 4.1 Radiomics for ICIP in NSCLC

Radiomics models can predict whether NSCLC will develop ICIP before immunotherapy, diagnose and differentiate patients with pneumonitis caused by immunotherapy, and predict the survival prognosis of NSCLC patients with ICIP.

#### 4.1.1 Radiomics for predicting the risk of ICIP in NSCLC

ICIP affects the survival rate and outcomes of patients with NSCLC. Early prediction of whether immunotherapy can cause pneumonitis in NSCLC patients and the selection of the most suitable candidates for immunotherapy can improve the survival outcomes of NSCLC patients. In 2016, Rivka R. Colen et al. (85) first used a radiomics model to predict ICIP (immunotherapy-induced pneumonitis). This study predicted the risk of ICIP in 32 cancer patients. Traditional radiomic methods were used to construct the predictive model, and CT images were segmented using 3Dslicer. A total of 1860 radiomic texture features based on histogram and Gray Level Co-occurrence Matrix (GLCM) were extracted. The MRMR method was applied to select the most relevant features for predicting ICIP. The method achieved a perfect accuracy of 100% (p=0.0033). However, due to the small number of cases in this study, the results may not be representative. Subsequently, Tan Peixin et al. (59) used a multimodal deep learning model to predict the risk of ICIP in 48 lung cancer patients (Including 47 cases of NSCLC), with an equal ratio of ICIP to non-ICIP patients. This study, for the first time, constructed a multimodal deep learning model for ICIP prediction based on nine clinical features and radiomic features. The deep learning model (3D ResNet18) employed in this study consists of 18 convolutional layers and corresponding fully connected layers. In contrast to conventional ResNet models, this model is specifically designed for processing three-dimensional data. The research employed two-stage transfer learning and contrastive learning strategies to enhance the performance of the prediction model. Ultimately, the model achieved an area under the curve (AUC) of 0.918 through five-fold cross-validation. M. Cheng et al. (86) also developed a multi-modal nomogram model, based on clinical and deep radiomics features, to predict the risk of ICIP in 141 lung cancer patients (Including 128 cases of NSCLC). The model’s predictions for ICIP were presented using a nomogram. To enhance the model’s performance and accuracy, they optimized the ResNet-50-V2 model using the Feature Pyramid Network (FPN). The study employed a random allocation method to divide patients into a training group (n=113) and a testing group (n=28). The results demonstrated that, both in the training group (0.910 vs. 0.871 vs. 0.778) and the testing group (0.900 vs. 0.856 vs. 0.869), the performance of the multi-modal nomogram model was superior to that of both radiomics and clinical models.

The toxic side effects of immunotherapy and radiotherapy overlap. The combination of immunotherapy and radiotherapy increases the incidence of pneumonitis (87). A KEYNOTE-001 study demonstrated that the risk of developing pneumonitis was higher with the combined use of immunotherapy and radiotherapy (63%) compared to using immunotherapy alone (40%) (88). Benjamin Spieler et al. (60) conducted an animal experiment in which Lewis lung carcinoma (LLC) cells were injected subcutaneously into 19 genetically identical (C57BL/6) mice. Among them, 15 mice received a combined treatment of radiation and immunotherapy, while the remaining 4 mice served as a control group. The mice in the treatment group were classified into two categories based on the median value of CD45 infiltration in the lungs. The experiment integrated pre-treatment radiomics features, NLR, and GM-CSF to construct a multimodal prediction model. CT and MRI imaging techniques were used, and 92 CT features and 92 MRI features were extracted. These features included geometric features, first-order histogram features, second-order joint probability features (such as co-occurrence matrix), and third-order joint probability features. The third-order joint probability features were developed by the researchers themselves. The experiment employed double cross-validation and demonstrated that the predictive performance of the model constructed based on MRI (AUC=0.834) was superior to that of the model constructed based on CT imaging (AUC=0.787). Linlin yang et al. (72) conducted a retrospective study and developed a Deep Graph Integrative Model (DG) to predict the risk of pneumonitis in NSCLC patients undergoing combined immunotherapy and radiotherapy. They employed two pre-trained CNN models (3D UNet) to extract features separately from tumor and lung volumes in the CT images. The extracted features were then selected and fused using the Graph Attention Layer (GAT) to construct the DG model. Using a five-fold cross-validation approach, the training set consisted of 195 cases (50 symptomatic pneumonitis), and the validation set consisted of 48 cases (12 symptomatic pneumonitis). The study results demonstrated that the DG model (AUC=0.823) outperformed conventional CT radiomics model (AUC=0.743), 3DUNet-based deep learning model (AUC=0.761), and DG models without GAT (AUC=0.796) (**Table 1** reports the cases mentioned in this paragraph).

**Table 1:** Summary of some radiomic studies for predicting cancer treatment-related pneumonitis.

Study	Study design	Modality	Treatment type	Number of prediction targets	Tumor Type	Features type	Features selection	Model algorithm	Type of validation
Colen et al. (2018) (85)	Retrospective Single-center	CT	ICI (n=32)	2	Cancer	Handcrafted radiomic	MRMR	Anomaly Detection Algorithm	LOOCV
Tan et al. (2022) (59)	Retrospective Single-center	CT	ICI (n=48)	24	Lung Cancer	Clinical; Deep radiomic	CNN	CNN	5FCV
Cheng et al. (2023) (86)	Retrospective Single-center	CT	ICI (n=141)	40	Lung Cancer	Clinical;Deep radiomic	CNN	CNN	Holdout
Spieler et al. (2022) (60)	Animal experiment	CT;MRI	RT+ICI (n=15)	8	Lewis	CBC; cytokine; Handcrafted radiomic	ANOVA	LR	2FCV
Yang et al. (2022) (72)	Retrospective Single-center	CT	RT+ICI (n=243)	62	NSCLC	Deep radiomic	CNN	DG	5FVC
Kawahara et al. (2021) (89)	Retrospective Single-center	CT	RT (n=77)	32	NSCLC	Handcrafted radiomic	LASSO	LR	5FCV

CT, Computed Tomography; ICI, Immune Checkpoint Inhibitor; MRMR, Minimum Redundancy Maximum Relevance; LOOCV, Leave-One-Out Cross Validation; CNN, Convolutional Neural Network; 5FCV, Five-Fold Cross Validation; MRI, Magnetic Resonance Imaging; RT, Radiation Therapy; CBC, Complete Blood Count; ANOVA, Analysis of Variance; LR, Logistic Regression; 2FCV, Two-Fold Cross Validation; NSCLC, Non-Small Cell Lung Cancer; DG, Deep Graph Integrative Model; LASSO, Least Absolute Shrinkage and Selection Operator.

#### 4.1.2 Radiomics for diagnosis and differential diagnosis of ICIP in NSCLC

In patients with NSCLC receiving immunotherapy, pneumonitis can be caused by factors other than immunotherapy. To determine whether Summary of some radiomic studies for predicting cancer treatment-related pneumonitis. is attributable to immunotherapy, some studies have used radiomics for the diagnosis of ICIP. The D. De Ruysscher team presented two studies on distinguishing ICIP and other causes of pneumonitis (OP) at the European Society for Medical Oncology (ESMO) in 2021 (90, 91). One study enrolled a total of 450 stage IV non-small cell lung cancer patients for immunotherapy treatment. CT imaging was performed six weeks after immunotherapy, and a total of 837 radiomics features were extracted from the images. Recursive Feature Elimination (RFE) was applied for feature selection, followed by a coherence analysis. Ultimately, 42 radiomics features with the highest correlation were selected. A logistic regression model was constructed using these features, achieving an AUC of 0.91 (95% confidence interval: 0.75 to 0.98). In another study, 556 stage IV NSCLC patients were recruited from six centers, with one center used for external validation. Clinical and radiomic features were used to construct clinical (AUC=0.99), radiomic (AUC=0.65), and combined models (AUC=0.99). The important predictive factors in the combined model are number of comorbidities, type of anti-PD(L)1 medication, dyspnea, and Gray Level Co-occurrence Matrix Inverse Difference Moment Normalized (GLCM IDMN) . In 2023, D. De Ruysscher et al. (92) published a retrospective study aimed at differentiating between ICIP and OP using clinical and radiomics features. They delineated seven different Regions of Interest (ROI) using either deep learning-based automatic segmentation or manual segmentation methods, and found that the radiomics model performed best when based on a 75mm spherical ROI. The first-order, Gray Level Co-occurrence Matrix (GLCM), Gray Level Run Length Matrix (GLRLM), Gray Level Size Zone Matrix (GLSZM), and Gray Level Dependence Matrix (GLDM) features were extracted from the CT images. An unsupervised approach, LASSO regression with 5-fold cross-validation, and stepwise backward logistic regression were employed to select radiomic features and construct the radiomics model, with model parameters being fitted using the original data. Through bootstrap validation, the performance of the radiomics model (AUC=0.83) was significantly superior to that of the clinical model (AUC=0.66).

Among treatment-related pneumonitis of NSCLC, the incidence of radiation therapy pneumonitis and ICIP is higher than that of pneumonitis caused by other treatments. Clinicians often face challenges in distinguishing between pneumonitis caused by radiation therapy and immunotherapy, making it difficult for them to determine the next course of treatment for NSCLC patients. There have been reports of using radiomics models to directly differentiate between radiation-induced pneumonitis (RIP) and ICIP in NSCLC patients (93, 94). Chen, X. G. et al. (93) retrospectively collected CT data from 82 NSCLC patients with pneumonitis (n = 23 after immunotherapy, n = 29 after radiotherapy, and n = 30 after combined immunotherapy and radiotherapy). The data from the immunotherapy and radiotherapy groups were used to train a random forest model, while the data from the combined immunotherapy and radiotherapy group were used for model testing. Seven radiomics features were selected using LASSO to construct the model, and hyperparameters were optimized using grid search. It was ultimately found that a machine learning model built solely on radiomics features could differentiate pneumonitis caused by ICI and RT in the training set (AUC = 0.76). The study also found that ICIP was more likely to occur bilaterally compared to RIP. Jun Cheng et al. (94) extracted three types of texture features from CT images, namely intensity histogram, gray level co-occurrence matrix (GLCM) based, and bag-of-words features. They constructed discrimination models using each of these three features. The radiomics model was built using datasets from patients receiving either immunotherapy or radiotherapy, and the dataset of combined immunotherapy and radiotherapy was employed for model testing. The discrimination model based on bag-of-words features achieved an AUC of 0.937 through 10-fold cross-validation. Qingtao Qiu et al. (95) conducted a retrospective study on 126 stage IV NSCLC patients with pneumonitis. They extracted 837 features from CT images and performed feature selection using LASSO. Eleven selected features were used to construct a nomogram model, which achieved an AUC of 0.901 in the validation set (**Table 2** reports the cases mentioned in this paragraph).

**Table 2:** Summary of some radiomic studies for pneumonitis diagnostic and differential diagnosis.

Study	Study design	Modality	Treatment Type	Tumor Type	Features type	Features selection	Model algorithm	Type of validation
Romita et al. (2021) (90)	Prospective Multi-center	CT	ICI (n=450)	IV stage NSCLC	Handcrafted radiomic	RFE	LR	External Validation
Tohidinezhad et al. (2021) (91)	Prospective Multi-center	CT	ICI (n=566)	IV stage NSCLC	Clinical; Handcrafted radiomic	——	LR	External Validation
Tohidinezhad et al. (2023) (92)	Retrospective Multi-center	CT	ICI (n=566)	IV stage NSCLC	Clinical; Handcrafted radiomic	Lasso	LR	Bootstrapping Validation
Chen et al. (2021) (93)	Retrospective Single-center	CT	RT (n=29); ICI (n=23); RT+ICI (n=30)	NSCLC	Clinical; Handcrafted radiomic	T-test; LASSO	RF	Holdout
Cheng et al. (2022) (94)	Retrospective Single-center	CT	ICI (n=28); RT (n=31); ICI+RT (n=14)	NSCLC	Clinical; Handcrafted radiomic	Two-Sided U Test	SVM	Holdout
Qing et al. (2022) (95)	Retrospective Single-center	CT	RT (n=79); ICI (n=57)	NSCLC	Clinical; Handcrafted radiomic	LASSO	LR	Holdout

CT, Computed Tomography; ICI, Immune Checkpoint Inhibitor; NSCLC, Non-Small Cell Lung Cancer; RFE, Recursive Feature Elimination; LR, Logistic Regression; LASSO, Least Absolute Shrinkage and Selection Operator; RF, Random Forest; SVM, Support Vector Machine; RT, Radiation Therapy.

#### 4.1.3 Radiomics for analyzing the prognosis of patients with ICIP in NSCLC

With the development of precision medicine, clinicians are increasingly focusing on personalized treatment for cancer patients.Currently, there is a large body of research on using radiomics models to predict the prognosis of immunotherapy in patients with NSCLC. Some studies incorporate clinical features on top of radiomics characteristics to enhance the accuracy of the prediction models (36, 96–102). Michael R. Sayer et al. (103) studied the impact of immune-related adverse events, relative occurrence time, and previous tyrosine kinase inhibitor (TKI) treatment on the performance of prognostic models for NSCLC patients treated with ICIs. The study found that Immune-related adverse events (irAEs) are important predictive factor for the survival prognosis of NSCLC patients. Therefore, in addition to predicting the prognosis of immunotherapy in patients with NSCLC, radiomics models can also be further utilized to predict the prognosis of NSCLC patients with ICIP.

### 4.2 Radiomics for other treatment-related pneumonitis in NSCLC

In order to achieve the optimal clinical benefits, patients with NSCLC are typically treated with a comprehensive treatment approach. However, radiotherapy (25), chemotherapy (26), targeted therapy (27, 104–106), and immunotherapy can all potentially lead to pneumonitis, which is known as treatment-related pneumonitis (107, 108). Patients who have received thoracic radiotherapy may experience radiation recall pneumonitis (RRP) after receiving anticancer drugs such as chemotherapy medications (109). Pneumonitis caused by taxanes (110) and platinum drugs is relatively rare. The overall incidence of targeted therapy-related pneumonitis is also low (1.2%) (106). The incidence of RRP induced by immunotherapy is 18.8% (with a median time of 368.5 days from the end of radiotherapy to the start of immunotherapy) (111). Radiomics models can predict and identify the treatment approaches that lead to pneumonitis in NSCLC patients, enabling early modification of the treatment plan to avoid or mitigate pneumonitis symptoms. Models constructed based on radiomics features and dose measurement variables can be used for predicting RIP in NSCLC patients. Studies have shown that a predictive model combining radiomics and dose measurement variables outperforms the use of radiomics alone (89, 112) (**Table 1**). Currently, dose measurement variables are considered important predictive factors for radiation-induced pneumonitis in NSCLC (113).

## 5 Conclusion and prospects

Currently, the radiomics models for NSCLC patients with ICIP primarily focus on diagnostic and differential diagnostic models. The predictive and prognostic analysis models for ICIP lack strong generalizability due to the complexity of cancer types used during model training. To improve model performance, some studies have incorporated highly relevant clinical features in addition to radiomics data. The accuracy of radiomics prediction remains insufficient, as immunotherapy is an emerging treatment approach with a limited number of NSCLC patients receiving immunotherapy, which is inadequate to support the development of a stable and high-performance radiomics model by computer algorithms. There is no gold standard for the diagnosis of ICIP, and clinical doctors primarily rely on patients’ clinical symptoms, pathogen examinations, and imaging manifestations for diagnosis. In the process of acquiring radiomics features, there is a lack of standardized protocols for slice thickness, imaging standards, and delineation of ROI in radiographic images, resulting in low reproducibility. To enhance the reproducibility of radiomics models, it is possible to construct an open-source radiomics data platform to compensate for the insufficient amount of data. The Cancer Imaging Archive (TCIA) is a large-scale cancer medical imaging archive hosting platform that allows for the download of open-source imaging data. The software tools ITK-SNAP (114), 3D Slicer (115), etc., can be used to delineate the ROI in radiological images. In order to establish standardized delineation criteria, software packages can be utilized for semi-automatic or fully automatic segmentation of the target area (116). Efficient and unified standards should be developed for each step of radiomics analysis. By doing so, the diagnostic and treatment process for NSCLC patients with ICIP will become more targeted and aligned with the principles of precision medicine.

## Author contributions

YS, WX and RS wrote the main manuscript content and revised it. PR prepared the tables for the manuscript. ZZ, LL and JZ assisted in editing the manuscript. ZC and GF designed the study, revised it, and gave final approval of the manuscript. All authors contributed to the article and approved the submitted version.
